# Improving engraftment of hepatocyte transplantation using alpha-1 antitrypsin as an immune modulator

**DOI:** 10.1007/s00109-019-01747-3

**Published:** 2019-02-28

**Authors:** Charlotte Lee, Anil Dhawan, Valeria Iansante, Celine Filippi, Ragai Mitry, Joanne Tang, Simon Walker, Raquel Fernandez DaCosta, Siddharth Sinha, Robin D. Hughes, Maria Koulmanda, Emer Fitzpatrick

**Affiliations:** 1 Dhawan Group at Mowat Labs, Institute of Liver Studies, King’s College London at King’s College Hospital, London, UK; 2 Paediatric Liver, GI and Nutrition Centre, King’s College London School of Medicine at King’s College Hospital, Denmark Hill, London, UK; 3 Departments of Medicine and Surgery, The Transplant Institute, Beth Israel Deaconess Medical Center/Harvard Medical School, Boston, MA USA

**Keywords:** Hepatocyte transplantation, Instant blood-mediated inflammatory reaction, Alpha-1 antitrypsin, Liver-based metabolic diseases

## Abstract

**Abstract:**

For patients with non-cirrhotic liver-based metabolic disorders, hepatocyte transplantation can be an effective treatment. However, long-term function of transplanted hepatocytes following infusion has not been achieved due to insufficient numbers of hepatocytes reaching the liver cell plates caused by activation of the instant blood-mediated inflammatory reaction (IBMIR). Our aim was to determine if the natural immune modulator, alpha-1 antitrypsin (AAT), could improve engraftment of transplanted hepatocytes and investigate its mechanism of action. A tubing loop model was used to analyse activation of the IBMIR when human hepatocytes were in contact with ABO-matched blood and 4 mg/ml AAT. Platelet and white cell counts, complement and cytokine expression were analysed. To determine if AAT could improve short-term engraftment, female rats underwent tail vein injection of AAT (120 mg/kg) or water (control) prior to the intrasplenic transplantation of 2 × 10^7^ male hepatocytes. At 48 h and 1 week, livers were collected for analysis. In our loop model, human hepatocytes elicited a significant drop in platelet count with thrombus formation compared to controls. Loops containing AAT and hepatocytes showed no platelet consumption and no thrombus formation. Further, AAT treatment resulted in reduced IL-1β, IL-6 and IFN-γ and increased IL-1RA compared to untreated loops. In vivo, AAT significantly improved engraftment of rat hepatocytes compared to untreated at 48 h. AAT infusion may inhibit the IBMIR, thus improving short-term engraftment of donor hepatocytes and potentially improve the outcomes for patients with liver-based metabolic disease.

**Key messages:**

• Alpha-1 antitrypsin (AAT) acts as an immune modulator to improve the efficacy of hepatocyte transplantation.

• Treatment with AAT decreased thrombus formation and pro-inflammatory cytokine expression in a tubing loop model.

• AAT significantly improved engraftment of donor hepatocytes within the first 48 h post transplantation.

**Electronic supplementary material:**

The online version of this article (10.1007/s00109-019-01747-3) contains supplementary material, which is available to authorized users.

## Introduction

Hepatocyte transplantation (HT) is a promising alternative to orthotopic liver transplantation (OLT) in paediatric patients with liver-based metabolic disorders [1–3]. The technique can be used as a bridge to organ transplantation or for effective long-term repopulation of the native liver, avoiding the need for liver transplantation. Liver transplantation is curative for certain liver-based metabolic disorders such as Crigler–Najjar syndrome (CNS), ornithine transcarbamylase (OTC) deficiency and maple syrup urine disease (MSUD); however, organs of suitable size and quality are rarely available at the time when the infant has the greatest need. These disorders, untreated, result in irreversible, neurological insult secondary to build up of toxic metabolic compounds as a consequence of a single enzyme deficiency. For example, in the case of neonatal onset ornithine transcarbamylase deficiency (OTC), the infant will suffer the effects of hyperammonaemia usually in the first few weeks of life and both appropriately sized organ availability and metabolic instability may preclude liver transplantation. Hepatocyte transplantation offers an immediately available and relatively less invasive alternative. The technique has shown a reduction in serum bilirubin by up to 50% in patients with CNS and a decrease in ammonia and an increase in urea production in patients with urea cycle defects [4–7]. However, despite numerous clinical cases reported, long-term clinical outcomes of hepatocyte transplantation have yet to be established with any type of liver-based metabolic disease. Poor long-term efficacy may be partly due to insufficient number of hepatocytes reaching the liver cell plates and significant cell loss has been observed within the first 24 h post transplantation, leading to poor engraftment and long-term cell function [8]. This has been attributed to an inflammatory reaction now commonly referred to as the instant blood-mediated inflammatory reaction (IBMIR), in which cells are recognised by the innate immune system, leading to rapid activation of both complement and coagulation pathways [9, 10]. Additionally, inflammatory cells including granulocytes, monocytes, Kupffer cells and natural killer (NK) cells are activated leading to rapid clearance and death of transplanted hepatocytes [11]. Alpha-1 antitrypsin (AAT) is a 52-kDa, 394 amino acid serine protease inhibitor produced by hepatocytes in the liver that inhibits a wide range of proteases including neutrophil elastase [12, 13]. AAT is a natural immune modulator with studies showing its anti-inflammatory and anti-apoptotic effects independent of protease inhibition [13, 14]. AAT directly affects the innate immune system through inhibition of protease-activated receptors (PARs), which leads to the inhibition of pro-inflammatory signalling cascades and cytokine release. Inflammatory cell recruitment is decreased, which further dampens the innate immune response [15]. Several animal studies have demonstrated the efficacy of AAT in improving islet graft survival and function [16–18]. In a similar manner to hepatocyte transplantation, islets are injected into the liver to provide continuing production of insulin for type 1 diabetic patients. Long-term efficacy and function of islet transplantation has yet to be demonstrated due to loss of graft function caused by activation of the IBMIR. A multi-centre review of autologous islet transplantation showed that 31% of islets lost function within 7 days and 50% of those that survived lost all function within a year [19]. In a syngeneic non-autoimmune islet graft mouse model, 2 mg of AAT per mouse, intravenously infused, maintained euglycaemia in 75% of recipients, compared to 20–27% in the controls. Furthermore, long-term function was demonstrated in a cynomolgus monkey islet transplantation model, in which AAT improved graft survival and function for up to 700 days post transplantation, compared to control monkeys in which blood glucose and C-peptide levels rose after just 80 days [18]. There are now several clinical trials in progress testing the use of AAT in the context of islet transplantation [20].

The aim of this study was to determine if AAT could inhibit activation of the IBMIR and improve engraftment in the context of hepatocyte transplantation. This was achieved by developing an in vitro tubing loop model and by using a wild-type rat model of hepatocyte transplantation.

## Methods

### Ethical approval

Ethical approval for the isolation and experiments involving primary human hepatocytes was obtained from the National Research Ethics Service (King’s College Hospital LREC 01-016). For blood donations from healthy volunteers, an amendment to these ethics was allowed (LREC 01-016 amendment 1 2014). Organs are donated through the National Health Service Blood and Tissue (NHSBT) and offered for hepatocyte transplantation following decline for solid organ transplantation with appropriate written consent in place for clinical and research use.

### Human hepatocyte isolation

Liver tissue that did not meet the criteria for clinical grade hepatocyte isolation and had appropriate consent for research purposes was processed in the liver research laboratory as described by Mitry et al. [21, 22]. Major blood vessels on the liver surface were cut, cannulated and secured through suturing. The tissue was perfused at a flow rate of 50–80 ml/min (size depending) with calcium-free HBSS, 4.6mM HEPES and 0.5 mM EGTA, followed by calcium-free HBSS and EMEM containing 0.05% collagenase P (Sigma-Aldrich, Dorset, UK). Once digested, the tissue was minced and sieved. Hepatocytes were purified by washing three times in ice-cold EMEM and centrifuged at 50×*g* at 4 °C for 5 min. Cells were cryopreserved at 1 × 10^7^ cells/ml in UW solution with 5% glucose and 10% DMSO using a controlled rate freezer (Kryo 10, series III, Planer Products, Ltd., Middlesex, UK) and stored at − 140 °C.

### Alpha-1 antitrypsin

Alpha-1 antitrypsin was provided by Professor Maria Koulmanda (Beth Israel Deaconess Medical Center/Harvard Medical School, Boston, MA). Alpha-1 antitrypsin (Aralast NP, Baxter, US Inc.) is prepared from large pools of human plasma and is treated with a solvent detergent to inactivate enveloped viral agents such as HIV, HBV and HCV. The half-life of AAT is reported to be between 3 and 5 days [23]. Aralast NP was supplied as a lyophilised powder and was freshly reconstituted in sterile water for injection and used within 3 h. The recommended human dosage is 60–120 mg/kg, which based on an average weight of 70 kg and average blood volume of 5 l is equivalent to 0.84–1.7 mg/ml of blood. Initial concentrations of 2 mg/ml and 4 mg/ml AAT were tested in the Chandler loop and compared to the controls of blood only, hepatocytes only and hepatocytes and 1 U/ml heparin (see supplementary material). For rat hepatocyte transplantations, 120 mg/kg AAT was used.

### Assessment of hepatocyte viability and function following AAT treatment

To determine if AAT (2–8 mg/ml) affected hepatocyte viability and function, MTT, albumin and urea assays were carried out. AAT was dissolved in 100 μl William's E (WE) medium and added to the cell culture media. Hepatocyte viability was determined using an MTT (3-(4,5-dimethylthiazol-2-yl)-2,5-diphenyltetrazolium bromide) assay. Briefly, cells were cultured for 24 h at 37 °C, with 5% CO_2_, supernatant was removed and cells were cultured with serum-free medium containing 0.5 mg/ml of MTT (Sigma-Aldrich, Dorset, UK) for 4 h. After removal of the MTT, the produced formazan was dissolved in DMSO and the optical density read at 570 nm on a Dynex MRX microplate reader. For albumin quantification, cell culture medium was collected 12 h post-plating and enzyme immunoassays carried out for human albumin (Bethyl Laboratories, Inc., TX, USA)*.* Ammonia metabolism was measured using a QuantiChrom™ Urea Assay kit *(*Universal Biologicals, Cambridge, UK). Cells were washed with PBS and incubated with 5 mM ammonium chloride (Sigma-Aldrich, Dorset, UK) for 6 h before measurement of urea synthesis.

### Chandler loop model

**Fig. 1:**
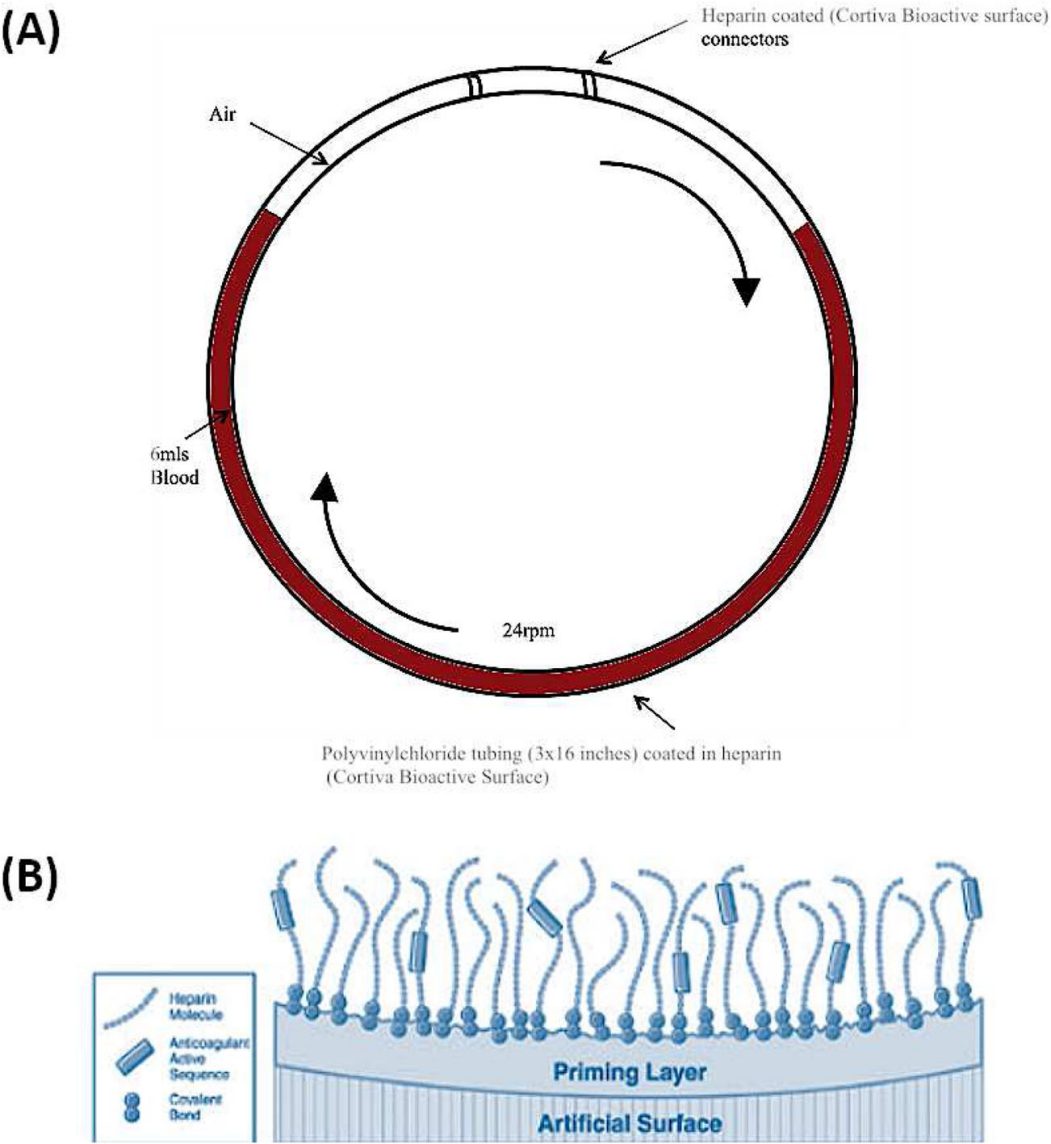
Chandler loop designed to mimic portal vein blood flow. **a** Polyvinyl chloride tubing (3 × 16 in.) was filled with 6 ml ABO-matched blood and hepatocytes. Loops were closed into circuits with heparin-coated polystyrene connectors. Tubing loops were rotated at 24 rpm and incubated at 37 °C for 0, 15, 30 and 60 min. **b** Schematic of Cortiva® BioActive Surface End Point Attached Heparin (http://www.medtronic.com/us-en/healthcare-professionals/products/cardiovascular/cardiopulmonary/cortiva-bioactive-surface.html). This method of anti-coagulation attaches heparin molecules via covalent bonds to amine groups on the prepared material surface. The aldehyde group on the heparin molecule is bound to the surface and the remainder of the molecule, including the active binding sequence is free to interact with the blood such as antithrombin

A Chandler loop model was developed to mimic portal vein blood flow and determine the effect primary human hepatocytes have on coagulation and complement parameters in ABO-matched human blood [24–26]. All human blood was obtained from healthy volunteers who had received no medication for at least 2 weeks prior to the experiments. Our final design of the Chandler loop system consisted of custom made polyvinylchloride (PVC) tubing (3 × 16 in.) coated in heparin using end-point attached heparin technology (Medtronic Cortiva® Bioactive surface, Watford, UK) (Fig. 1). PVC tubing was filled with 6 ml ABO-matched blood and 5 × 10^6^ primary human hepatocytes suspended in 500 μl of CMRL transplant media. Loops were closed into circuits with heparin-coated polystyrene connectors. Tubing loops were rotated at 24 rpm and incubated at 37 °C using a mini tube rotator. Blood samples were taken at 0, 15, 30 and 60 min into 1 ml ethylenediaminetetraacetic acid (EDTA) (1.8 mg EDTA/ml of blood) blood tubes.

Whole blood samples were immediately analysed for full blood counts on an ADIVA 2120 haematology system (Siemens Healthcare Diagnostics, Surrey, UK)*.* The remaining samples were centrifuged at 2000×*g* for 15 min to obtain the plasma. Samples were stored at − 80 °C until analysis of inflammatory cell cytokines and complement factor C5b-9. Results shown are *N* = 6 (six different batches primary human hepatocytes and six different blood donors).

### Cytokine analysis

Plasma samples collected from tubing loop experiments were stored at − 80 °C without any freeze/thaw cycles and analysed for cytokine expression using a high sensitivity Randox HS X Biochip Array designed to measure 12 cytokines/chemokines (IL-2, IL-4, IL-6, IL-8, IL-10, VEGF, IFN-γ, TNF-α, IL-1α, IL-1β, MCP-1) (Randox Laboratories Ltd., County Antrim, UK)*.*

### Electron microscopy

Electron microscopy was carried out at the UltraStructual Imaging Department, King’s College London. Thrombi that formed within the Chandler loop were fixed in 2.5% glutaraldehyde in 0.1 M cacodylate buffer at 4 °C overnight. Samples were washed in 0.1 M cacodylate buffer and left at 4 °C until tissue processing. Samples were incubated with 1% osmium tetroxide at 4 °C for 30 min and washed three times in wash buffer. Samples were progressively dehydrated in 20%, 50%, 70%, 90% and 3 × 100% ethanol for 5 min. Samples were transferred to fresh 100% ethanol and critical point dried using carbon dioxide in a Leica critical point dryer. Samples were mounted on stubs with conductive carbon cement (TAAB) and sputter coated with gold. Samples were examined and recorded using a FEI Quanta 200F field emission scanning electron microscope operated at 35 kV in high vacuum mode.

### Immunohistochemistry

Thrombi were embedded in OCT medium, snap-frozen in liquid nitrogen and stored at − 80 °C. Thrombi were sectioned on a cryotome (6-μm slices) and stained with a monoclonal mouse anti-human hepatocyte antibody Clone OCH1E5 (Dako, Cambridge, UK) and a secondary goat anti-mouse HRP antibody (Invitrogen, Paisley UK) and visualised using DAB+ substrate and chromagen (Dako, Cambridge, UK). Slides were stained with Shandon Instant Haemotoxylin and counterstained with 1% eosin for visualisation of nuclei and cytoplasm.

### Animals

All animals were handled following protocols approved by the Ethical Review Process of King’s College London in accordance with the UK Animals (Scientific) Procedures Act of 1986. Animals were housed under 12-h light/dark cycles in a room maintained at 21 ± 2 °C and 55 ± 10% humidity in the Denmark Hill Biological Services Unit (BSU). Food and water were available ad libitum*.* Animals were left to acclimatise for at least 1 week before any procedure.

### Primary rat hepatocytes

Hepatocytes were isolated from 250 to 350 g Sprague Dawley rats (Charles River, Harlow, UK), by in situ collagenase perfusion of the liver as previously described [27]. The cell suspension was centrifuged at 50×*g* for 5 min at 4 °C to isolate the hepatocytes. Cell viability was determined using trypan blue and always reached at least 65%. Cells were cryopreserved at 1 × 10^7^ cells/ml in UW solution with 5% glucose and 10% DMSO and thawed at time of transplant [28]. To allow cell tracking, the transplanted cells were stained with 60 μM CM-DiL, a lipophilic carbocyanine cell tracker dye, excitation 553 nm, emission 570 nm (Thermo Fisher Scientific, Paisley, UK). Cells were incubated with the CM-DiL dye at 37 °C for 5 min and then at 4 °C for 15 min. Unbound dye was washed off by centrifugation at 50×*g* for 5 min. In addition, eGFP rat hepatocytes were isolated from Lewis-Tg (CAG-eGFP) Ysrrc rats and are known as HepaCur™. The cells were kindly donated by Yecuris, Portland, OR, USA*.*

### Hepatocyte transplantation

Wild-type Sprague Dawley rats 250–350 g (Charles River, Harlow, UK) (*N* = 3/time point) were anaesthetised using isoflurane (5% induction and 2.5% maintenance) and their temperature maintained at 37 °C using a heating pad. A midline laparotomy was performed to transplant 15 × 10^6^ CM-DiL or eGFP-labelled hepatocytes re-suspended in CMRL transplant media (PAN Biotec, Dorset, UK) via the spleen using a 23 G needle. Following removal of the needle, bleeding was stopped using spongostan film. For post-operative analgesia, the rats received 0.02 mg/kg buprenorphine immediately after the procedure subcutaneously and 0.13 ml/kg of meloxicam (Loxicom) 1 day post procedure via oral administration.

### AAT administration

Rats underwent administration of 120 mg/kg of AAT or water for injection (controls) via the tail vein before intrasplenic hepatocyte transplantation. Lyophilised AAT was freshly reconstituted in water for injection just before infusion. AAT was re-administered at day 0, 3, 7 and 10.

### Haematology analysis

Rat blood samples were taken before, 0 h, 24 h, 48 h and 1 week post transplantation via the tail vein. Whole blood was immediately analysed for full blood counts on an ADIVA 2120 haematology system (Siemens Healthcare Diagnostics, Surrey, UK)*.* Prothrombin time was taken before, 1 h, 48 h and 1 week post transplantation using a Coagucheck XS meter and Coaguchek XS PT strips (Roche Diagnostics Limited, West Sussex, UK)*.* Human AAT levels in rat plasma were measured using a Human AAT ELISA quantification kit (Bethyl Laboratories, Inc., TX, USA)*.* The optical density was read at 450 nm on a Dynex MRX microplate reader.

### Identification of engrafted hepatocytes

#### CM-DiL-labelled rat hepatocytes

Animals were euthanised at 48 h and 1 week. To analyse the effect of AAT on cell engraftment, the amount of CM-DiL present in the rat liver was quantified. The IVIS Lumina Series III imaging machine (Perkin Elmer, Buckinghamshire, UK) was used to track transplanted cells in the whole liver ex vivo. Due to significant auto-fluorescence, the animals could not be imaged in vivo*.* Transplanted hepatocytes labelled with CM-DiL dye were imaged with an excitation of 560 nm and emission 620 nm with the exposure time set to 2 s. A large proportion of CM-DiL positive cells were macrophages that had incorporated the lipophilic dye. Therefore, to determine the number of engrafted hepatocytes in rat liver sections, analysis required co-staining with the pan macrophage antibody CD68. Slides were counterstained for CD68 (Abcam, Cambridge UK) at a 1:500 dilution for 1 h at RT, followed by a donkey anti-rabbit Alexa-fluor 488 secondary antibody (Thermo Fisher Scientific, Paisley, UK) at a 1:200 dilution for 1 h at RT. Slides were counterstained with DAPI diamond mounting media (Thermo Fisher Scientific, Paisley, UK)*.* Rat liver sections were cut from three of the major rat liver lobes: middle lobe (ML), left lateral lobe (LLL) and right lobe (RL). Two sections from each liver lobe were analysed per rat, making a total of six sections/rat and the entire section imaged on an Inverted Microscope Leica DMi8 (Leica Microsystems UK Ltd., Milton Keynes, UK) using the tile scanning feature. For analysis, a co-localisation plugin (ImageJ, National Institute of Heath, Maryland, USA) was used to quantify the number of double-stained cells in the whole section, which was removed from the total number of positive CM-DiL cells. The number of single-stained CM-DiL hepatocytes was expressed as a percentage of the total number of cells in the section (assessed using the nuclear DAPI staining).

#### eGFP-labelled rat hepatocytes

To further confirm engraftment, eGFP cells were quantified within liver sections. Animals were euthanised at 24 h, 48 h, 1 week and 1 month after transplantation. Liver tissue was fixed in 10% formal saline and paraffin embedded and cut (4-μm sections) by the Institute of Liver Studies Pathology department. Slides were counterstained with DAPI diamond mounting media (Thermo Fisher Scientific, Paisley, UK) and imaged on an inverted Microscope Leica DMi8 (Leica Microsystems UK Ltd., Milton Keynes, UK) using the tile scanning feature. The number of eGFP hepatocytes was expressed as a percentage of the total number of cells in the section (DAPI).

### Immunohistochemistry

Rat liver paraffin sections were stained with the trichrome stain Martius Scarlet Blue. Fibrin was stained with crystal scarlet solution, collagen was stained with methyl blue and red blood cells were stained by picric acid. Sections were stained courtesy of the Liver Histopathology department, Institute of Liver Studies, King’s College Hospital. Slides were imaged on a Leica DFC/7000 T Microscope × 200 total magnification. For identification of tissue factor, cells were stained with a primary rabbit monoclonal tissue factor antibody at a 1:50 dilution for 1 h at RT, followed by a goat anti-mouse/rabbit dual HRP antibody (Dako, Aligent, Santa Clara, USA) at a 1:200 dilution for 1 h at RT. Slides were counterstained with haematoxylin.

### Detection of transplanted hepatocytes by PCR analysis for the Y chromosome gene (SRY)

To detect the presence of male donor cells engrafted in the female livers, genomic DNA was extracted from the female recipient livers 48 h and 1 week post transplantation using the DNeasy blood and tissue kit prior to qPCR for the SRY (sex-determining region Y) gene, which is only present in male cells. Primer sequences designed for the SRY gene were forward (fwd) 5′-CGAAGGGTTAAAGTGCCACAG-3′ and reverse (rv) 5′-GTTCTTGGAGGACTGGTGTGC-3′ (Thermo Fisher Scientific, Paisley, UK) [29]*.* 5-HTT was used as a control housekeeping gene. Primer sequences for the 5-HTT gene were fwd 5′-TCCGCATGAATGCTGTGTAAC-3′ and rev 5′-TTGGCTTAGAGGGGAGGAGTC-3′ (Thermo Fisher Scientific, Paisley, UK) [29]*.* This control gene was used to quantify total genomic DNA.

A series of dilutions of male genomic DNA was used to construct standard curves (333.3 ng/μl–8.25 pg/μl). The percentage of donor male genomic DNA in female recipient samples was determined by dividing the amount of male DNA by the total genomic DNA (5-HHT). Both female rat hepatocytes and sham liver samples were used as a negative control. The PCR programme was 95 °C for 15 min, 40 cycles of 94 °C for 15 s, 57 °C for 30 s and 72 °C for 30 s as described by Xue et al. [29] and was run on the Applied Biosystems QuantStudio 7 Flex Real Time PCR system (Thermo Fisher Scientific, Paisley, UK)*.*

### Statistical analysis

Normality was tested for using the Kolgrov–Smirnov normality test. Data were evaluated using two-way repeated measures ANOVA with a Bonferroni post hoc test. qPCR data for the SRY gene were analysed using a non-parametric Mann–Whitney *U* test. All data are expressed as mean ± SEM.

## Results

### Primary human hepatocytes trigger the IBMIR in an in vitro blood perfusion model

**Fig. 2:**
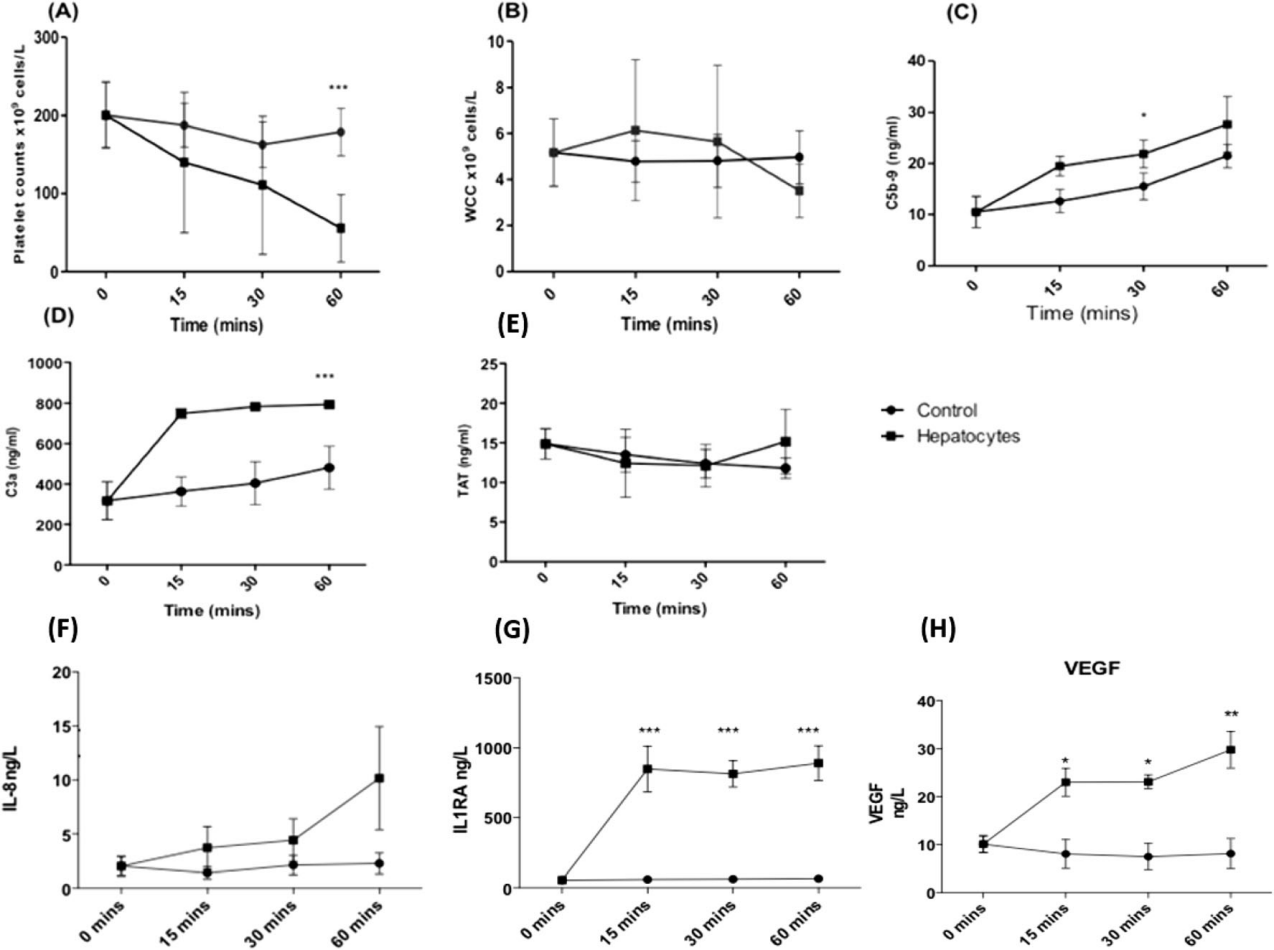
Primary human hepatocytes activate the IBMIR in an in vitro tubing loop model containing ABO-matched blood. **a** Platelet count. **b** White cell count. **c** C5b-9. **d** C3a. **e** Thrombin-antithrombin. A Randox high sensitivity X Biochip array was used to measure expression **f** IL-8, **g** IL-1RA and **h** VEGF. *N* = 6 (platelet and WCC), *N* = 3 (C5b-9, C3a, TAT, IL-8, IL1RA and VEGF), values are mean ± SEM *P < 0.05, ***P < 0.001

Human hepatocytes were added to the Chandler loop tubing containing ABO-matched blood and samples taken at 0, 15, 30 and 60 min. After 60 min, the presence of hepatocytes led to a significant drop in platelet count (55.5 × 10^9^ cell/l ± 17.6) compared to control blood without hepatocytes (179 × 10^9^ cell/l ± 12.5) (Fig. 2a, *N* = 6, *P* < 0.001). After 60 min, there was a lower white cell count (WCC) in blood samples containing hepatocytes (3.5 × 10^9^ cell/l ± 0.47) compared to control samples (5.12 × 10^9^ cell/l ± 0.60) (Fig. 2b, *N* = 6). Hepatocytes triggered a significantly higher C5b-9 expression at 30 min (21.9 ng/ml ± 2.7) and a higher C3a expression at 60 min (794.2 ng/ml ± 5.7) compared to control samples (15.3 ng/ml ± 2.6 and 481 ng/ml ± 61, respectively) (Fig. 2c, d; *N* = 3, *P* < 0.05). There was no significant difference in thrombin-antithrombin (TAT) production after 60 min between control and hepatocyte loops (11.8 ng/ml ± 0.75 vs 15.1 ng/ml ± 2.4) (Fig. 2e; *N* = 3, *P* > 0.5).

Plasma from Chandler loop samples was analysed for cytokine expression (IL-2, IL-4, IL-6, IL-8, IL-10, VEGF, IFN-γ, TNF-α, IL-1a, IL-1β, MCP-1). After 60 min, hepatocytes triggered an increased expression of IL-8 (Fig. 2f; 2.3 ± 1.0 ng/l vs 10.2 ± 4.8 ng/l, N.S), IL-1RA (Fig. 2g; 29.8 ± 3.8 ng/l vs 890.6 ± 124 ng/l, *P* < 0.001) and VEGF (Fig. 2h; 8.2 ± 3.2 ng/l vs 30.0 ± 3.8 ng/l, *P* < 0.001) compared to control blood. There was no significant effect on IL-2, IL-4, IL-6, IL-10, IFN-γ, IL-1A, IL-1β and MCP-1 (*N* = 3, N.S).

**Fig. 3:**
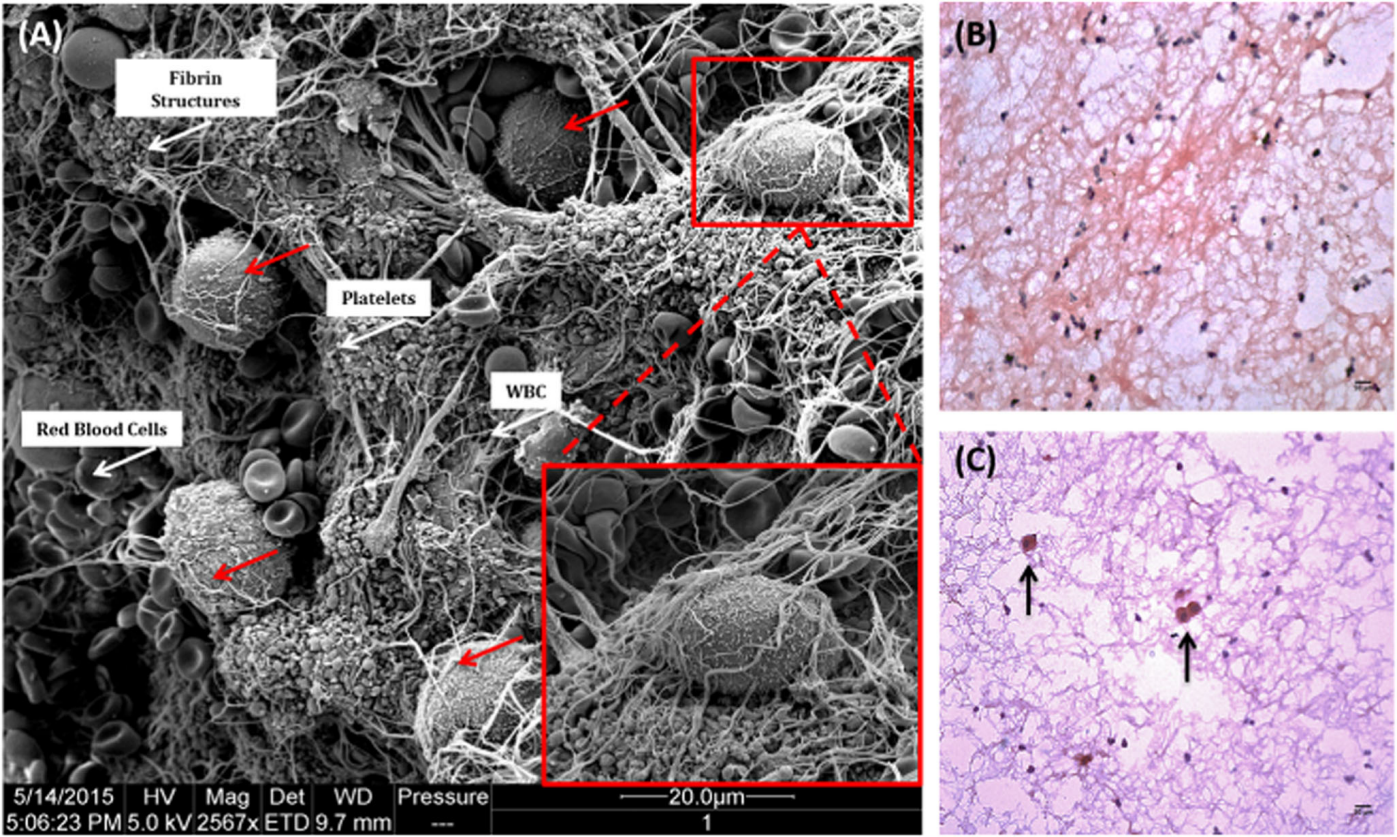
Primary human hepatocytes trigger large thrombus formation when in contact with ABO-matched blood. **a** Thrombi that formed within the Chandler loop model were fixed in 2.5% glutaraldehyde and prepared for electron microscopy. Hepatocytes became entrapped within thrombi, shown by red arrows and magnified inset image. Red blood cells, white blood cells, platelets and fibrin deposits are all visible within the thrombus (white arrows). Samples were examined and recorded using a FEI Quanta 200F field emission scanning electron microscope operated at 35 kV in high vacuum mode. Eight micrometre sections were cut and stained with a monoclonal mouse anti-human hepatocyte antibody (Clone OCH1E5), followed by a dual HRP polymer secondary antibody and counterstained with haematoxylin and eosin. **b** Thrombus from control loop containing blood only. **c** Thrombus from loop containing ABO-matched blood and hepatocytes. × 100 total magnification

After 60 min in contact with ABO-matched blood in the Chandler loop model, hepatocytes triggered large thrombus formation within these loops. Thrombi were fixed in 2.5% glutaraldehyde and analysed using scanning electron microscopy. Images revealed numerous hepatocytes embedded within these thrombi which were identifiable by their large size and microvilli on the cell surface (Fig. 3a). This was further confirmed in cryosections using a human hepatocyte-specific antibody (Clone OCH1E5) (Fig. 3b, c).

### Effect of AAT on primary human hepatocyte viability and function

**Fig. 4:**
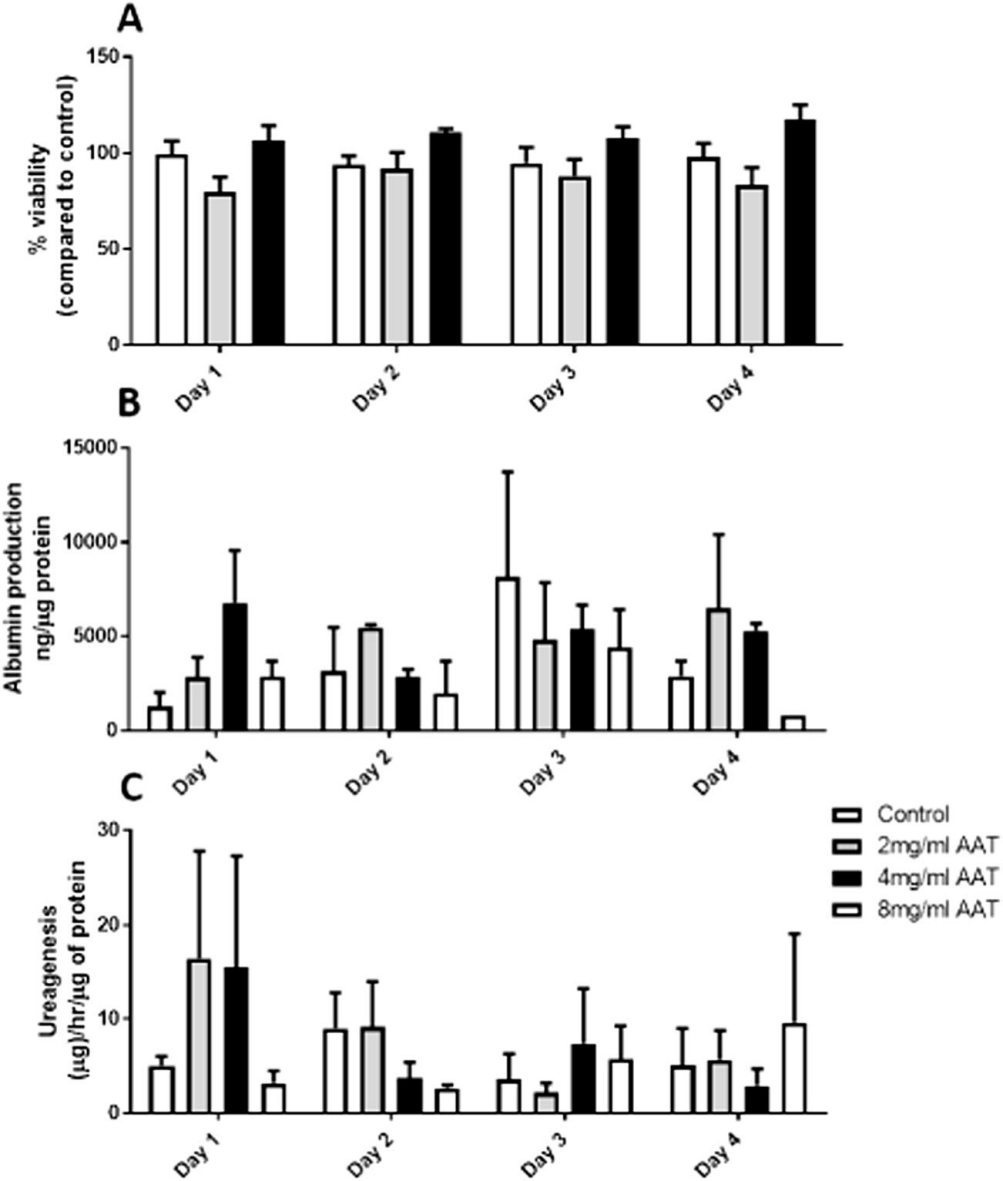
The effect of AAT (2–8 mg/ml) on primary human hepatocyte viability and function over a 4-day period. **a** Cell viability assessed using MTT assay. **b** Albumin production measured by ELISA. **c** Ureagenesis of hepatocytes treated with AAT measured using a QuantiChrome Urea assay. *N* = 3, N.S.

An MTT assay was used to assess primary human hepatocyte viability following treatment with increasing concentrations of AAT (2 to 8 mg/ml). Hepatocyte viability was not compromised by 2–8 mg/ml of AAT (Fig. 4a; *P* > 0.05, *N* = 3). At day 1, there was a trend towards increased albumin and urea production from hepatocytes treated with 4 mg/ml AAT but this did not reach significance (Fig. 4b, c; *P* > 0.05, *N* = 3). Importantly, 2–8 mg/ml did not significantly inhibit hepatocyte function (Fig. 4b, c; *P* > 0.05, *N* = 3).

### Effect of AAT to inhibit the IBMIR

**Fig. 5:**
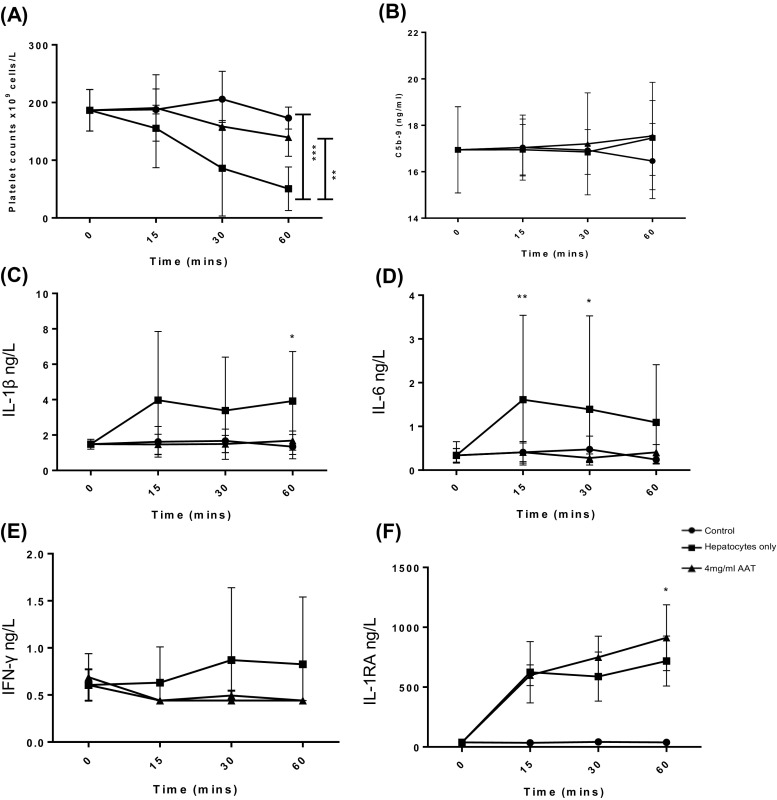
The effect of 4 mg/ml AAT on IBMIR activation by primary human hepatocytes in the Chandler loop model. 5 × 10^6^ human hepatocytes were added to 6 ml of ABO-matched human whole blood and rotated at 24 rpm at 37 °C for 1 h to mimic portal blood flow. **a** Platelet count *N* = 6. **b** C5b-9 expression *N* = 6. A Randox high sensitivity X Biochip array was used to measure expression of **c** IL-1β, **d** IL-6, **e** IFN-γ and **f** IL-1RA, *N* = 3. *P < 0.05, **P < 0.01, ***P < 0.0001

Increasing concentrations of AAT were tested using the Chandler loop model to determine the optimal dose to inhibit the IBMIR (2–4 mg/ml). Human hepatocytes suspended in CMRL transplant media were added to ABO-matched blood and AAT in the loop. This was compared to the standard use of 1 U/ml of heparin, which is added to CMRL transplant media in current clinical hepatocyte transplantation protocols. Four milligramme per millilitre AAT was the most effective concentration to inhibit platelet consumption compared to loops containing hepatocytes, 1 U/ml heparin and 2 mg/ml AAT (see supplementary material). Four milligramme per millilitre AAT significantly reduced platelet consumption compared to loops containing hepatocytes alone (Fig. 5a; 156 × 10^9^ vs 62 × 10^9^ cell/l, *N* = 6, **P < 0.01, ***P < 0.001). C5b-9 expression was not affected by 4 mg/ml AAT after 60 min in the loop (Fig. 5b; *P* > 0.05). Following 60 min in the Chandler loop model, 4 mg/ml of AAT prevented increased expression of pro-inflammatory cytokines that was observed with hepatocytes only—IL-1β (1.7 vs 3.9 ng/l, IL-6 (0.4 vs 1.1 ng/l) and IFN-γ (0.4 vs 1.3 ng/l), achieving similar levels to control blood-only IL-1β (1.3 ng/l), IL-6 (0.24 ng/l) and IFN-γ (0.44 ng/l (Fig. 5c–e; *N* = 3, *P* < 0.05). At the same time, 4 mg/ml AAT significantly increased concentrations of the anti-inflammatory cytokine IL-1RA compared to the control only loop (Fig. 5f; 912.9 vs 37.8 ng/l, *N* = 3, *P* < 0.05).

### Intrasplenic rat hepatocyte transplantations

**Fig. 6:**
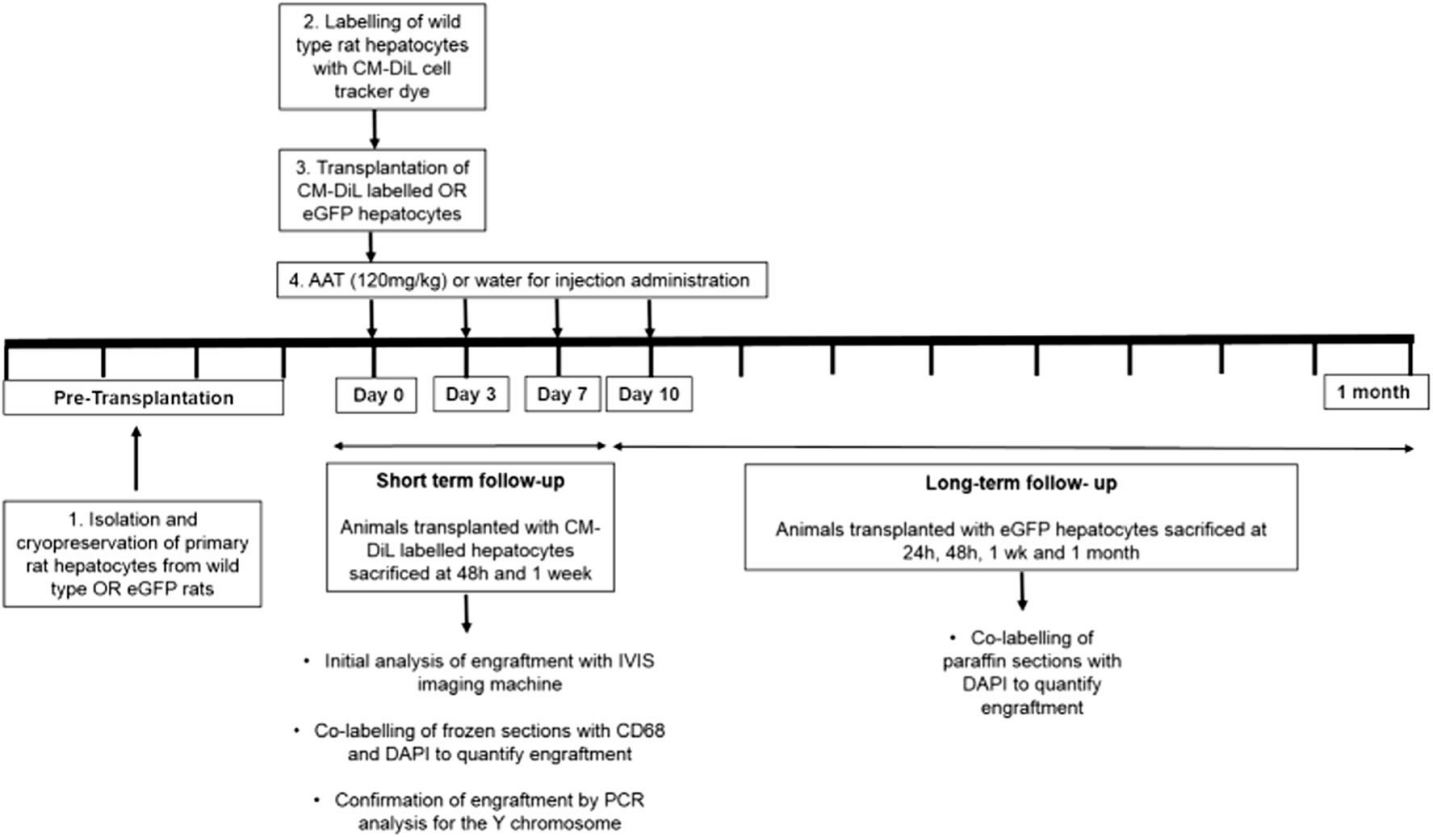
Experimental design to study the effects of alpha-1 antitrypsin on hepatocyte engraftment. Primary rat hepatocytes were isolated from either Sprague Dawley wild-type (WT) rats or eGFP Wistar rats and cryopreserved. WT cells were labelled with the cell tracking dye CM-DiL. 15 × 10^6^ hepatocytes were transplanted intrasplenically following the IV injection of 120 mg/kg AAT or water for injection (controls). Animals that received CM-DiL-labelled cells were sacrificed at 48 h and 1 week and engraftment quantified in the entire liver using the IVIS imaging machine. Further analysis was carried out in frozen liver sections by co-staining with DAPI and CD68 to ensure macrophages that engulfed transplanted hepatocytes were not included in the analysis. Analysis was further confirmed by PCR analysis for the Y chromosome (SRY). For long-term engraftment, rats received eGFP hepatocytes and engraftment was quantified in paraffin sections by co-labelling with DAPI

**Fig. 7:**
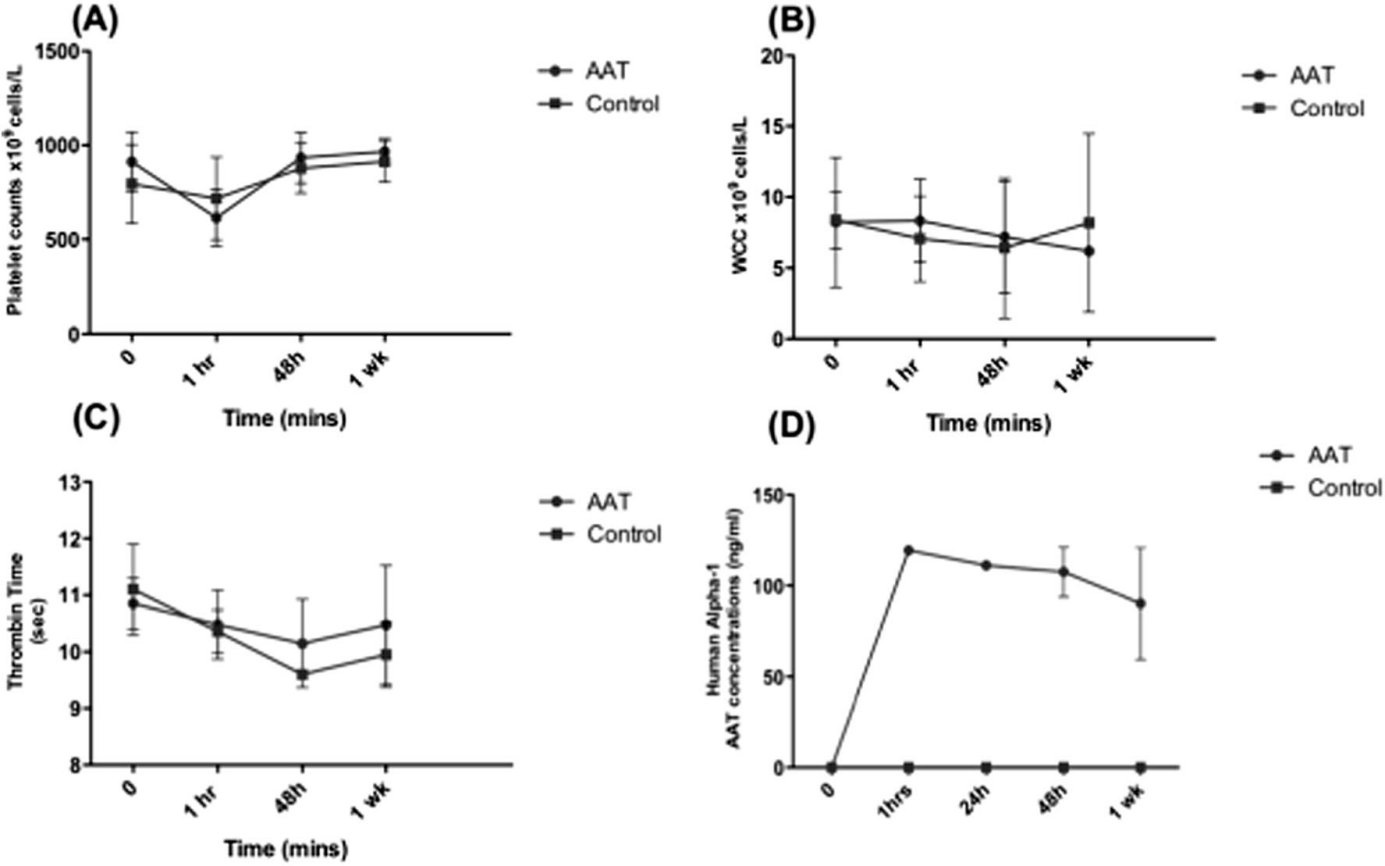
The systemic effects of AAT on Sprague Dawley rats 0 h (pre-transplantation), 1 h, 24 h, 48 h and 1 week post transplantation. **a** Platelet count. **b** White cell count. **c** Thrombin time. 0 h, 1 h, 48 h and 1 week *N* = 5. **d** Systemic human alpha-1 antitrypsin concentration, 0 h, 1 h, 24 h, *N* = 1, 48 h and 1-week *N* = 3, N.S.

Wild-type Sprague Dawley rats were used to test the effect of AAT on hepatocyte engraftment (Fig. 6). Average viability of donor hepatocytes upon isolation was 86% with an average yield of 5.28 × 10^8^cells per liver. Hepatocytes were cryopreserved using standard protocols and thawed at the time of transplantation. AAT (120 mg/kg Aralast NP) or water for injection (controls) was administered via the tail vein before hepatocytes (average viability 75%) were transplanted intrasplenically. Blood samples were taken before transplantation, and at 1 h, 48 h and 1 week post transplantation. There was no significant difference in platelet count, white cell count and thrombin time between control and AAT treated rats (Fig. 7a–c). The concentrations of human alpha-1 antitrypsin were measured in the plasma to monitor metabolism over time following IV injection every 3 days. Concentrations of human AAT were maintained at 1 h, 24 h, 48 h and 1 week post transplantation (Fig. 7d). There was no human AAT found in control rats or pre-transplantation samples showing no cross reactivity with endogenous rat AAT production.

**Fig. 8:**
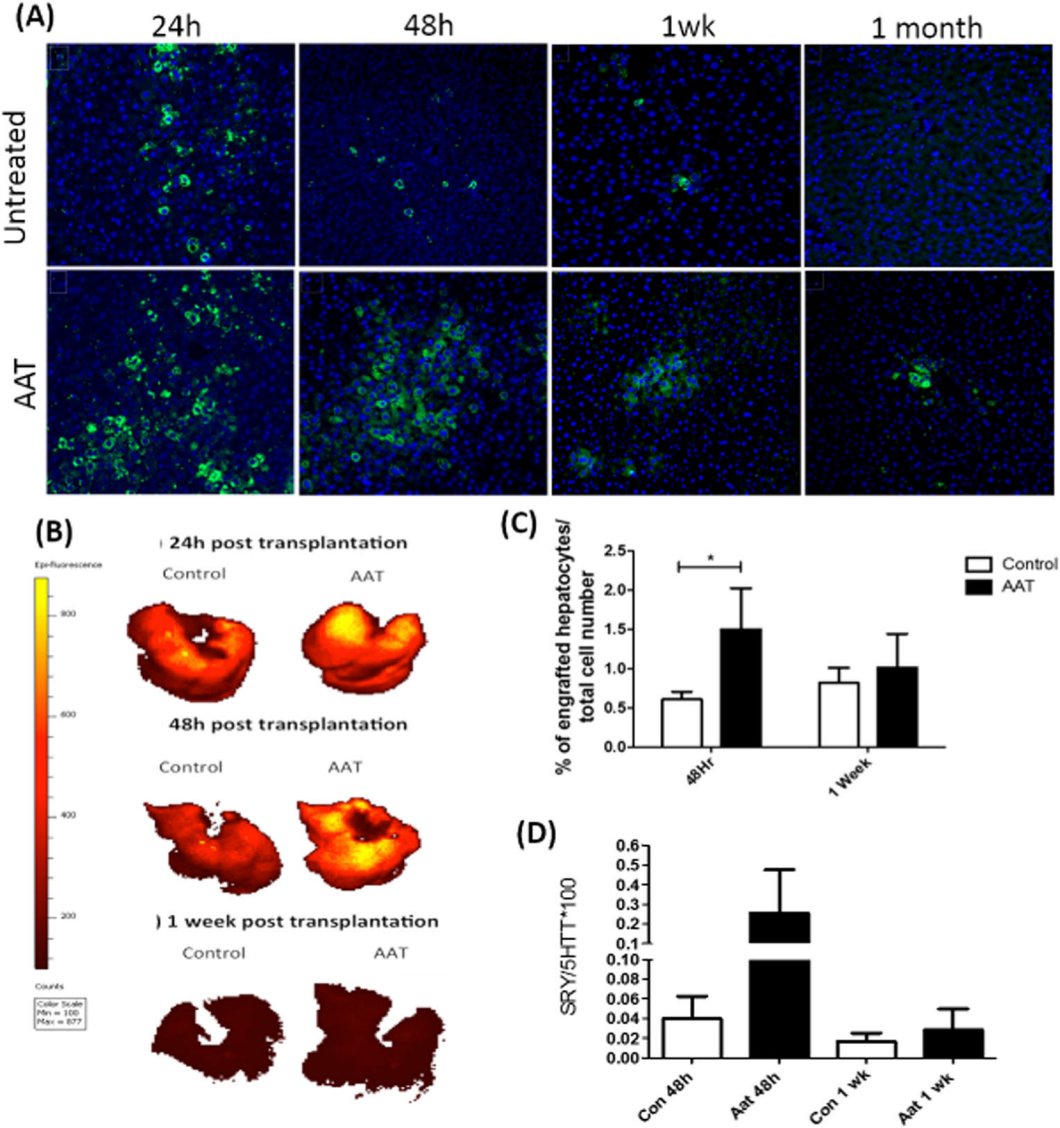
The effect of AAT on engraftment of eGFP rat hepatocytes at 24 h, 48 h and 1 week post transplantation. Sprague Dawley rats underwent intrasplenic transplantation of 15 × 10^6^ eGFP-labelled hepatocytes. **a** Paraffin sections were labelled with DAPI mountant media. Blue represents the DAPI nuclear stain and the green is the eGFP transplanted hepatocytes. Images were taken on an Inverted Microscope Leica DMi8 × 200 total magnification (Leica Microsystems UK Ltd, Milton Keynes, UK)*.***b** Representative images of rat livers ex vivo showing the fluorescent measurement of CM-DiL-labelled transplanted hepatocytes in the liver of control and AAT-treated rats. Images were taken at 24 h, 48 h and 1 week post transplantation using an IVIS Spectrum in vivo Imaging System. Number of transplanted cells is proportional to counts with the colour scale from red (low signal/low cell engraftment) to yellow (high signal/high cell engraftment). **c** Engraftment of hepatocytes is expressed as the percentage of engrafted cells over the total number of cells (DAPI). **d** Genomic DNA was extracted from female recipients at 48 h and 1 week following intrasplenic transplantation of male donor cells. qPCR analysis for the SRY gene was carried out using Applied Biosystems QuantStudio 7 Flex Real Time PCR system (Thermo Fisher Scientific, Paisley, UK). *N* = 3 per time point. *P < 0.05

To monitor the effect of AAT on CM-DiL-labelled hepatocyte engraftment, the liver was removed and imaged ex vivo on the IVIS imaging system. There was a higher CM-DiL signal in the AAT-treated rats compared to controls at 24 h and 48 h as shown by the epi-fluorescence count scale (Fig. 7b). There was low CM-DiL signal after 1 week in both control and AAT-treated rats. To further analyse engraftment of CM-DiL-labelled cells, frozen sections were used. However, following co-staining with the macrophage antibody CD68, a significant number of CM-DiL cells were macrophages that had engulfed transplanted hepatocytes and incorporated the lipophilic dye. As a result, the number of engrafted hepatocytes was quantified by counterstaining with the macrophage marker CD68. Following co-localisation analysis, it is possible to distinguish between transplanted hepatocytes and macrophages that have engulfed hepatocytes and more accurately quantify the effect of AAT on hepatocyte engraftment. This method confirmed that AAT significantly increased hepatocyte engraftment at 48 h (Fig. 8c; 48 h 1.6 ± 0.3% vs 0.6 ± 0.1%, *N* = 3 *P* < 0.05). No statistical significance was seen at 1 week between treatment and control groups (Fig. 8c; 1.2 ± 0.3% vs 0.8 ± 0.2%, *N* = 3, *P* > 0.05). To further confirm improved hepatocyte engraftment with AAT, experiments were repeated using GFP-labelled cells and engraftment tracked at 24 h, 48 h, 1 week and 1 month which similarly showed increased engraftment in AAT-treated animals at 24 h and 48 h (Fig. 8a; 24 h; 4.5 ± 1.4% vs 1.0 ± 0.2%, **P < 0.01 and 48 h 2.1 ± 0.5 vs 1.0 ± 0.3%, *P < 0.05).

To further quantify the effect of AAT on hepatocyte engraftment, DNA was extracted from female recipient rats and PCR analysis used to quantify the Y chromosome gene (SRY) present in transplanted male donor cells. At 48 h, there was a 10-fold increase in the percentage of male positive cells in the AAT group compared to controls, but not reaching significance (Fig. 8d; 0. 3 ± 0.2% vs 0.04 ± 0.02%, *N* = 3, *P* > 0.05). At 1 week, there was an increase in the percentage of male positive cells in the AAT group compared to controls, but this was not significant (Fig. 8d; 0.028 ± 0.021% vs 0.012 ± 0.007%, *N* = 3, *P* > 0.05).

### AAT alters tissue factor and fibrin expression

**Fig. 9:**
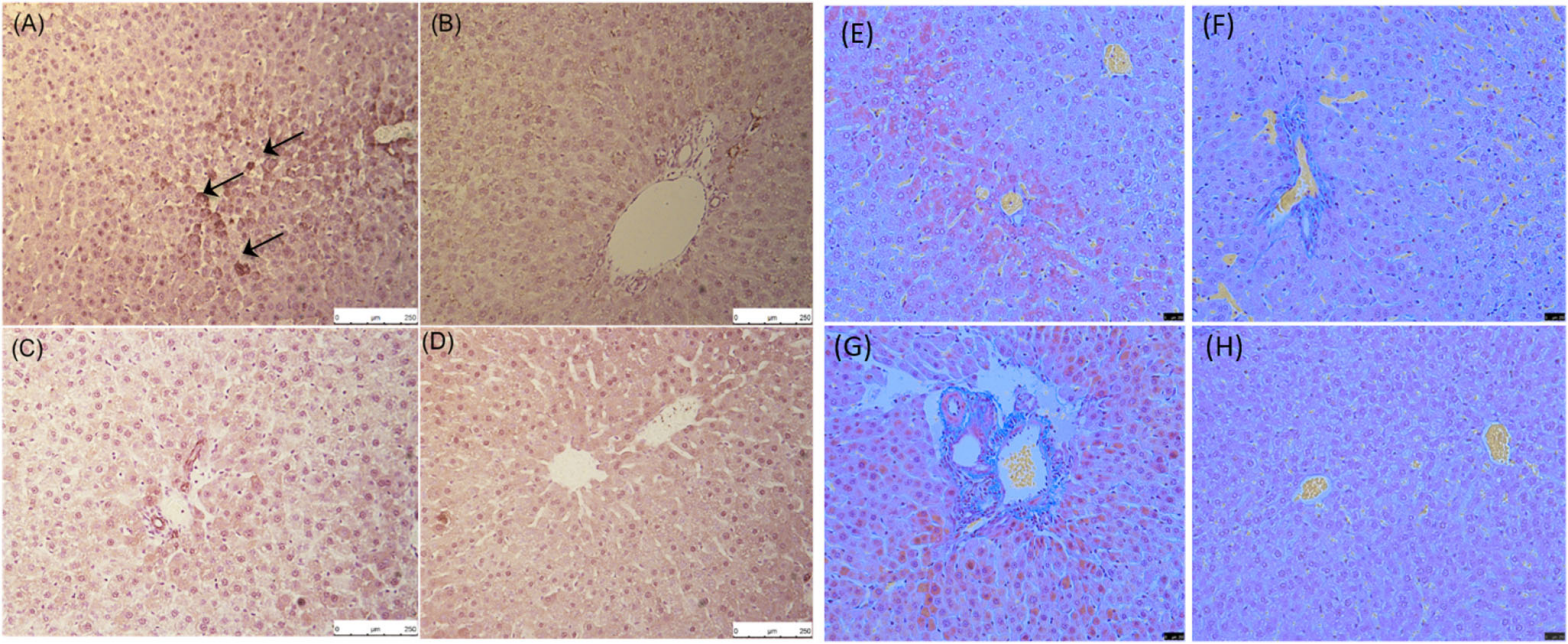
Representative images of rat liver sections stained with a tissue factor and Martius Scarlet Blue. Tissue factor: **a** control 48 h post transplantation. **b** AAT 48 h post transplantation. **c** Control 1 week post transplantation. **d** AAT 1 week post transplantation. Martius Scarlet Blue Fibrin is stained in red, collaged in blue and red blood cells in yellow. **e** Control 48 h post transplantation. **f** AAT 48 h post transplantation. **g** Control 1 week post transplantation. **h** AAT 1 week post transplantation. Images were taken on a Leica DFC/7000 T Microscope × 200 total magnification. Images are representative of staining carried out on three different rats/condition. Arrows indicate positive staining

The IBMIR has been well described to be activated by tissue factor (TF) produced by hepatocytes [30, 31]. To further investigate the effect of AAT on coagulation activation, rat liver sections were stained with a tissue factor antibody. AAT-treated rats had minimal TF expression at 48 h and 1 week. Control rats had strong TF expression in rat liver sections at 48 h but this was not evident at the 1-week time point (Fig. 9). To determine if the lack of TF expression led to a decrease in fibrin formation, Martius Scarlet Blue staining was performed. Images show AAT-treated rat liver sections have fewer fibrin deposition than controls at both 48 h and 1 week (Fig. 9).

## Discussion

AAT is a promising natural immune modulator with several studies demonstrating anti-inflammatory and anti-apoptotic properties. The aim of this study was to investigate if AAT could inhibit the IBMIR in models of hepatocyte transplantation. In an in vitro tubing loop model, a dose of 2–8 mg/ml of AAT did not affect human hepatocyte viability and function and was sufficient to decrease coagulation activation in a Chandler loop model. In this model, 4 mg/ml AAT inhibited the increased concentration of inflammatory cell cytokines IL-6, IL-1β and IFN-λ whilst maintaining the increased concentration of the anti-inflammatory cytokine IL-1RA. Extensive image analysis of CM-DiL-labelled and eGFP hepatocytes showed 120 mg/kg AAT significantly increased hepatocyte engraftment at 48 h in a WT rat transplantation model. This was further demonstrated by qPCR analysis for the Y chromosome gene.

This is the first study to use clinical grade PVC tubing and connectors coated in heparin using end-point attached heparin technology for a Chandler loop model (Cortiva™, Medtronic Carmeda® Bioactive surface, Watford, UK). The heparin coating is designed to specifically mimic the vascular endothelium layer and is supported by a large amount of peer-reviewed clinical and scientific evidence showing its biocompatibility [32]. Using this model, a dose of 4 mg/ml was sufficient to prevent the platelet depletion that is observed when hepatocytes are added to ABO-matched blood alone. This suggests AAT may inhibit coagulation activation. Although a clear role of AAT on thrombosis has not been established, many of the activated clotting factors are serine proteases including factor X, XI, XII, thrombin and plasmin. Therefore, the ability of AAT to inhibit serine proteases may provide a link between inflammation and thrombosis [33]. AAT has also been shown to inhibit the serine proteases that are involved in cytokine release through PAR (protease-activated receptors) inactivation [13]. In the Chandler loop model AAT directly decreased concentrations of IL-6, IL-1β and IFN-γ, which is consistent with previously published studies [15]. In this model, hepatocytes did not significantly increase TAT concentrations as previously shown [9]. This is hypothesised to be due to the type of heparin coating used. This tubing is designed with end-point attached heparin that preserves the active site of immobilised heparin so it can interact with blood elements such as antithrombin. The resulting heparin-antithrombin complex has a 1000× greater affinity for coagulation factors such as thrombin compared to antithrombin alone. As a result, although large clot formation was observed in loops containing hepatocytes, the TAT complexes may still be attached to the immobilised heparin that is coating the tubing and were therefore not detectable using an ELISA. Further work is required to find a marker that more accurately reflects coagulation activation in this model that is unaffected by the heparin coating in the tubing.

In Sprague Dawley rats, administration of 120 mg/kg AAT had no systemic effect on platelet count, WCC and thrombin time, suggesting no significant harmful effects. The safety and efficacy of AAT has been previously shown in six clinical trials that have investigated the use of 60–120 mg/kg of AAT in the field of islet transplantation [34]. Currently, the dose of 60 mg/kg used in clinical trials for islet transplantation is based on augmentation therapies for patients with AAT but was not based on pre-clinical data in the context of islet transplantation. It has now been shown that in islet transplantation, a short descending dose protocol of 240 mg/kg, 120 mg/kg and 60 mg/kg over 7 days is advantageous over the traditional 21 days of treatment with 60 mg/kg [35]. Furthermore, dividing the original 60 mg/kg dose into 3 × 20mg/kg injections was superior in improving both circulating AAT levels and islet graft survival. Further investigations are still required to determine the optimum dose of AAT in the context of hepatocyte transplantation.

To determine the long-term effects of AAT in hepatocyte transplantation and to investigate its anti-inflammatory effects on hepatocyte engraftment, intrasplenic hepatocyte transplantations were carried out in wild-type Sprague Dawley rats. To initially track cell engraftment the cell tracker dye CM-DiL was used. However, this dye was incorporated into macrophages at all time points. To more accurately quantify CM-DiL-labelled hepatocytes, a CD68 pan-macrophage marker was used to differentiate between transplanted hepatocytes and Kupffer cells that might have engulfed the transplanted cells. Using this method, AAT significantly increased engraftment after 24 h and 48 h but not at 1 week. Markus et al. transplanted CM-DiL rat hepatocytes directly into liver lobules and showed using FACS analysis that the number of labelled hepatocytes decreased over time from 2.1% after 24 h to 0.5% on day 10, which is comparable to our results [36]. In our study, increased cell engraftment was further confirmed using eGFP hepatocytes and by quantitative PCR analysis for the Y chromosome gene. These results were comparable to image analysis results, showing a greater percentage of transplanted hepatocytes at 48 h in the AAT group compared to the control. Previous studies using combined liver and liver cell transplantation in Lewis rats, following 2-acetylaminofluorene (2-AAF) and partial hepatectomy, showed at day 8 there was 0.15% of male DNA which rose to 1.2% at day 15 and 14.2% at 90 days. Potentially, pre-conditioning of the host liver may have increased the percentage of male transplanted hepatocytes. It has been reported that liver re-population and long-term survival and function requires a combination of proliferative stimuli to the transplanted hepatocytes and suppression of host hepatocytes [37].

To investigate the effect of AAT on coagulation activation, tissue factor production was analysed. At 48 h post transplantation, control rat liver sections showed tissue factor staining within the parenchyma, which was inhibited by AAT. As coagulation factors are serine proteases, it could be that AAT inhibits factor VIIa activation and therefore TF production. Decreased tissue factor expression was associated with decreased fibrin formation in rat liver sections. Staining with Martius Scarlet Blue showed fibrin deposition in control rat livers, which was absent in AAT-treated animals. This is comparable to our Chandler loop data, which showed that AAT inhibited platelet consumption, suggesting an inhibition of coagulation activation and fibrin formation. In an islet transplantation model, AAT was shown to reduce fibrinogen deposition on islet grafts, which is detrimental to their survival [38]. These results suggest the potential antithrombotic effects of AAT and its ability to inhibit the IBMIR.

We have shown AAT significantly improved engraftment at 24 h and 48 h but were unable to show an effect at 1 week and 1 month. This study is limited due to the lack of liver pre-conditioning. As a result, the donor hepatocytes had no selective advantage to engraft over endogenous hepatocytes. Previous studies have demonstrated that long-term hepatocyte proliferation and survival requires a combination of proliferative stimuli and suppression of host hepatocytes. This is typically done using partial hepatectomy which involves a 60–80% removal of the liver, and retrorsine treatment to inhibit endogenous hepatocytes [37, 39]. The lack of liver pre-conditioning is one of the major limitations of this study. However, such treatment is far removed from clinical application, so the results would need to be interpreted carefully. Furthermore, this study has not been able to demonstrate the long-term benefits of AAT at 1 week and 1 month due to the lack of immunosuppression, which was avoided to enable investigation into the effects of AAT on the immune system. As a result, cell-mediated rejection led to clearance of transplanted cells at longer time points. Current clinical hepatocyte transplantation protocols use corticosteroid methylprednisolone, based on liver transplantation protocols. Further work is required to investigate the interaction of AAT and steroidal immunosuppressant’s to determine whether they may enhance proliferation and survival of hepatocytes or whether the combination may inhibit its beneficial effects.

This study has shown AAT may inhibit activation of the IBMIR in vitro and may improve short-term engraftment in a wild-type animal model. However, to further validate the efficacy of AAT in improving hepatocyte transplantation, the Gunn rat model may be particularly useful, in which a naturally occurring single guanosine base deletion within the UGT1A1 gene results in a lack of enzyme activity and severe hyperbilirubinemia [40]. The genetic defect in Gunn rats closely mimics the clinical symptoms observed in Crigler–Najjar patients and is therefore an accurate pre-clinical model for demonstrating improved cell function with AAT.

## Conclusion

This is the first study to investigate the potential of the natural immune modulator AAT to inhibit the IBMIR in the context of hepatocyte transplantation. AAT has been widely investigated in several clinical trials to date suggesting its safety and efficacy. We have used a novel in vitro blood perfusion model to show 4 mg/ml AAT prevented platelet consumption and activation of pro-inflammatory cytokines observed when hepatocytes were in contact with ABO-matched blood. In a wild-type rat model of hepatocyte transplantation, AAT prolonged hepatocyte graft survival at 48 h compared to untreated controls. AAT decreased platelet depletion, fibrin formation and TF production suggesting its ability to inhibit coagulation activation. Further work using a model of metabolic liver disease is required to determine if AAT can improve hepatocyte function and long-term engraftment before clinical application can be considered.

## Electronic supplementary material

Supplementary Figure 1 (PNG 92 kb)

High resolution image (EPS 171 kb)
