# Supplementary figures and images for: Improving engraftment of hepatocyte transplantation using alpha-1 antitrypsin as an immune modulator
Source: J Mol Med (Berl). 2019 Feb 28;97(4):563–77. doi: 10.1007/s00109-019-01747-3 (PMC6440943; doi:10.1007/s00109-019-01747-3)

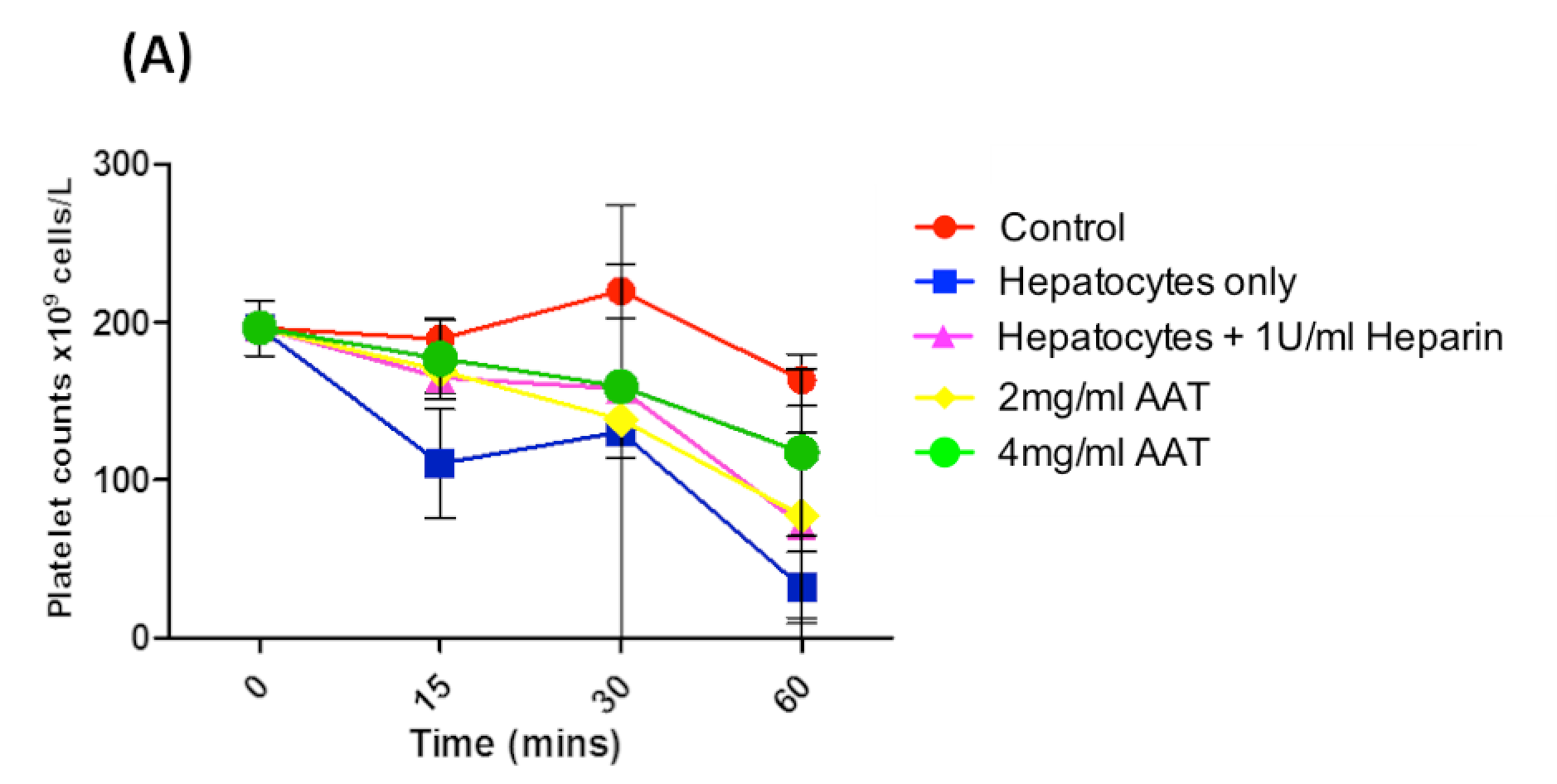

Supplement: Supplementary file 1 — (PNG 92 kb) [file 109_2019_1747_Fig10_ESM.png]
